# A longitudinal analysis of injury characteristics among elite and amateur tennis players at different tournaments from electronic newspaper reports

**DOI:** 10.3389/fpubh.2022.835119

**Published:** 2022-08-10

**Authors:** Rabiu Muazu Musa, Isyaku Hassan, Mohamad Razali Abdullah, Mohd Nazri Latiff Azmi, Anwar P. P. Abdul Majeed, Noor Azuan Abu Osman

**Affiliations:** ^1^Centre for Fundamental and Continuing Education, Universiti Malaysia Terengganu, Kuala Nerus, Terengganu, Malaysia; ^2^Faculty of Languages and Communication, Universiti Sultan Zainal Abidin, Gong Badak Campus, Kuala Nerus, Terengganu, Malaysia; ^3^East Coast Environmental Research Institute Universiti Sultan Zainal Abidin, Kuala Nerus, Terengganu, Malaysia; ^4^Innovative Manufacturing, Mechatronics and Sports Laboratory, Faculty of Manufacturing Engineering, Universiti Malaysia Pahang, Pekan, Malaysia; ^5^School of Robotics, XJTLU Entrepreneur College (Taicang), Xi'an Jiaotong-Liverpool University, Suzhou, China; ^6^Centre for Applied Biomechanics, Department of Biomedical Engineering, Faculty of Engineering, University of Malaya, Kuala Lumpur, Malaysia; ^7^Chancellery, Universiti Tenaga Nasional, Kajang, Malaysia

**Keywords:** data mining, elite and amateur players, injury analysis, media content analysis, tennis game, electronic newspapers

## Abstract

The non-complexity of tennis, coupled with its health benefits, renders it appealing and encourages varying competitions at different levels of age, gender, and expertise. However, the rapid increase in the participation rates witnesses a surge in injury occurrences, prompting the need for in-depth analysis to facilitate immediate intervention. We employed a media content analysis technique in which tennis-associated articles published in the last 5 years were examined. A total of 207 news reports were gathered and screened for analysis. Subsequently, 71 articles were excluded from the study due to content duplications or summary updates of existing news articles, while 23 news articles were also excluded from the study due to inappropriateness. Finally, 113 news reports directly related to injury in tennis were coded and analyzed. We examined various types of injuries reported from the screened articles with respect to their status (fresh, recurrent, and recovery) across expertise levels i.e., elite, or amateur. Similarly, the incidence of injury occurrences based on the types of tournaments the players engage in was also investigated. A chi-square analysis was employed to achieve the objectives of the study. Occurrences of tennis-associated injuries are disseminated across expertise levels [$\chi_{\left(18\right)}^{2}$ = 16.542; *p* = 0.555], with knee, hip, elbow, and shoulder injuries being highly prevalent in both elite and amateur players. Nevertheless, it was noted that elite players suffered a staggering 72.60% of injury-related problems, while amateur players sustained 27.40% of injuries. Moreover, the status of injury spreads based on types of tournaments [$\chi_{\left(4\right)}^{2}$ = 3.374; *p* = 0.497], with higher occurrences of fresh and recurrent injuries, while low recovery rates were observed. The findings further demonstrated that injuries are sustained regardless of tournament types [$\chi_{\left(36\right)}^{2}$ = 39.393; *p* = 0.321]. However, most of the injuries occurred at international tournaments (85%). Whereas, only 5.30% of the injuries occurred at national/regional tournaments while 9.70% were unidentified. It could be deduced from the findings of this investigation that elite players are more prone to injuries compared with amateur players. Furthermore, the most common tennis-related injuries affect the lower, trunk, and upper regions of the body, respectively. A large number of the reported tennis injuries are fresh and recurrent, with a few recoveries. The international tennis tournaments are highly attributed to injury occurrences as opposed to the national/regional tournaments. The application of the media-based data mining technique is non-trivial in projecting injury-related problems that could be used to facilitate the development of an injury index peculiar to the tennis sport for prompt intervention.

## Introduction

Tennis is amongst the most famous individual and non-contact sports in the world, with around 1.17 percent of the world's population participating in the sport (1). The non-complexity of tennis, coupled with its health benefits, renders it appealing and encourages varying competitions at different levels of expertise, age, and tournaments. Unlike many other sports, there is no time limit on how long players can play during a match. Consequently, matches can extend for hours, requiring numerous rallies driven by powerful surges of energy (2). The constant need for both aerobic and anaerobic energy to enable the execution of many types of strokes has led to the development of a variety of injury indexes (3). Moreover, recent data have demonstrated a startling growth in the frequency of injury occurrences during both training and competitions and thus, warrant an in-depth analysis for an urgent intervention (4, 5).

Systematic research is needed to better identify tennis-associated injuries and to develop effective ways to avoid them (4, 6, 7). The compilation of appropriate and reliable data for tennis injury assessments, on the other hand, continues to be a difficult task (8). Furthermore, effective injury data gathering methods are necessary to enable extensive research of tennis-related injuries (9). In this context, studies have demonstrated that mainstream media, particularly newspapers, are important in offering helpful data for injury analysis (10, 11). Newspapers have been observed to report more injury-associated occurrences as compared to other media (11). Injury occurrence could become newsworthy and a matter of interest to the audience. As a result, newspapers can be a source of injury information (12, 13).

Additionally, existing literature has demonstrated the significance of newspaper content as a robust and reliable source of information (14–16). Previous investigation has shown that newspapers represent a dynamic information source from which practitioners and researchers can obtain a variety of data to support decision-making. Thus, valuable information about tennis-related injuries could be gathered from newspapers to support medical professionals in developing preventive measures to curtail the prevalence of tennis-related injuries at different levels of participation (16).

Moreover, abundant and comprehensive tennis injury data could be collected from electronic newspapers. This is because, compared with specialized global sports media that typically pay attention to performance and outcomes, electronic newspapers are more likely to report tennis-related injury occurrences locally and globally (17, 18). As such, this study considered Nigerian newspapers as data sources due to their print and online presence, which allows them to cover local, regional, and international sports events to reach a global audience (19). Also, tennis is a very vibrant sport in Nigeria with high popularity among different age groups (20).

Though earlier studies examined tennis-associated injuries using various methodologies (6, 8, 21), the authors noted the difficulties in collecting easily available and relevant injury data that might be used for analysis to guide decision-making. Additionally, none of the previous studies concentrated on a media-based data mining examination of tennis-related injuries. Likewise, a major lack of injury-related data has been documented in developing nations (11, 22). As a result, this study analyses tennis-related injuries acquired from chosen Nigerian electronic newspapers between January 2015 and December 2020 using media content analysis. The newspapers were chosen based on their online readership and reputation. To the best of our knowledge, this study represents an early attempt to evaluate injury occurrences in tennis from the content of mainstream media. The study looks specifically at injury incidence and exposure to tennis-related injuries based on players' levels of experience and tournament types. It is worth highlighting that the present study employed quantitative content analysis to investigate injury prevalence in tennis. This approach could potentially assist coaches and relevant stakeholders to address injury-related data paucity in curtailing the prevalence of injury in tennis. In particular, the methodological approach proposed to achieve the objectives of this study has been successfully applied to analyze health-related issues and proved to be highly reliable (23, 24).

### Motivation for the research

Tennis represents a popular sport that promotes a variety of tournaments for all categories of age, gender, and experience due to its simplicity and health advantages. The popularity of the sport witnessed a surge in overall participation globally. A recent report by the International Tennis Federation (ITF) projected that, currently, more than 87 million players are actively playing the sport across over 41 nations, which contributed to an increase in participation rate by 4.5 percent in the year 2020 compared with 2019 data obtained from 195 countries (25). The report further indicated significant growth in the number of tennis courts, clubs, and coaches globally. In another development, a recent report published by the Physical Activity Council's Participation (26) shows an increase in tennis participation by 22 percent in the US alone during the COVID-19 pandemic in 2020, with over 21.64 million people hitting the courts.

Nevertheless, the sharp rise in participation rates coincides with an increase in injury incidents. Recent studies on competitive adolescent tennis players revealed the prevalence of injury occurrences of 1.2 to 2.8 injuries per 1,000 h played (27, 28). Moreover, a significant number of complaints emanated from the junior tennis players, while many of such injuries are found to be recurrent, with a weekly increase rate of 12.1 percent in comparison with a 3 percent rise in acute injury incidents (8). To mitigate the lingering problem of tennis-related injuries, several techniques have been introduced, such as the application of a hand-held dynamometer as well as a smartphone inclinometer as part of the measures for injury detection, prevention and return to play (29, 30). A more recent study investigated the efficacy of an e-health monitoring system in the prevention of tennis-specific injury using a randomized control trial technique amongst adult recreational players (31). It was demonstrated from the findings of the study that the provision of an un-supervised e-health tennis-specific exercise programme was unable to reduce the occurrence of injuries and therefore should not be employed.

Despite such efforts, the rate of injury occurrences remains high. It is worth noting that the high rate of injury occurrences in tennis does not only affect the career of athletes but also has significant economic impacts on both athletes and concerned authorities (32). Despite these problems, comprehensive programs for tennis injury prevention are either lacking or inadequate (33). As such, research has indicated the need for systematic investigation to recommend useful strategies for the prevention and management of tennis-related injuries (4, 6). Hence, in the current investigation, we seek to carry out an in-depth analysis of tennis-related injuries to facilitate immediate intervention. An in-depth analysis of injury occurrences across various tournaments and levels of participation could potentially assist in discovering the common injury sites in tennis players, which can be used as a strategy for injury prevention by strength and conditioning specialists *via* a tailored training and exercise at the affected regions.

## Materials and methods

### Content analysis technique

This study adopts a content analysis to examine tennis-related injuries in five major Nigeria-based online newspapers. Content analysis was chosen due to its ability to explore the characteristics of media messages. This is because content analysis provides specific media content that can be analyzed to produce useful information. This method also looks at communication *via* texts and allows for both quantitative and qualitative analyses (34, 35). Content analysis is identified as a research method based on facts, as opposed to other methods such as discourse analysis (36). Also, content analysis “takes texts and analyses, reduces and interrogates them into summary form through the use of both pre-existing categories and emergent themes to generate or test a theory” (37).

### Sampling procedure and period of coverage

Five Nigerian newspapers, i.e., *Vanguard, Punch, The Nation, The Sun, and ThisDay*, were chosen for examination. These national newspapers have the highest readership and online popularity. They also represent the most widely circulated daily English language periodicals (Top 10 Nigerian Newspapers, 2019). News items focusing on tennis-related inquiries were obtained from the respective digital archives of the selected newspapers between January 2015 and September 2020. This era corresponds to a period when different tennis competitions, such as the Rio Olympics, Wimbledon Championships, Australian Open, and US Open, are held throughout the world. The time range was chosen to reduce the data to a size that could be easily analyzed. Due to their presence on both print and digital platforms, Nigerian newspapers were selected for this study. Because they are available in numerous formats, the newspapers, therefore, can reach a global audience and cover local, international, and regional sports news (19).

### Variables search

The terms “Tennis” and “Injury” were used as keywords to search for relevant news articles. The content analysis comprised all of the news items discovered using this strategy. For analysis, only straight news and feature stories were chosen. There were a total of 113 articles found to be relevant. Since readers may access online news at any time (38, 39), online news articles were considered in this study. Newspapers are also regarded as a more thorough and trustworthy data source than traditional datasets (11, 12). Although news content is considered qualitative, it may be processed to yield measurable findings. As a result, content analysis is mostly regarded as a blend of both qualitative and quantitative research techniques (34). Similarly, news coverage has shown to be a good source of information on the occurrence and magnitude of injury-related problems (12).

### Method of coding and reliability assessment

At first, each publication in the original sample was reviewed to determine its relevancy based on headlines and news content. A codebook was developed by the researchers using a hierarchical code system to guide the coding process as proposed by Hsieh and Shannon (34). In the codebook, statistical occurrences for each item were ascertained using predetermined codes. The *codes* are structured as a hierarchy of codes where the top-level *codes* represent the research variable while the bottom-level *codes* represent categorical variables, followed by descriptions and exemplary quotes for each code from the news articles. In this sense, each variable is not limited to having a single category. Coding disagreements were deliberated, and subsequently, the coding scheme was reviewed and used in the study (see Appendix 1). The hierarchical coding system is beneficial because it can express a large quantity of information in a small number of digits (34). Consequently, a database was created using Excell with five categorical variables: Injury locations, tournament types, as well as the level of expertise which was determined based on players' age. Accordingly, players aged 8–17 years were categorized as amateurs whilst those aged 18 years and above were considered as elites (40, 41). To guarantee uniformity, inter-coder reliability was employed. The level of inter-coder agreement was ascertained by means of Cohen's kappa test.

Furthermore, the news stories were cross-checked to eliminate the double-entry of data. Subsequently, duplicates or summary updates of existing articles were excluded from the study. Additionally, emerging themes were verified to avoid reproducing the news content. Similarly, the emerging themes were validated to prevent replicating media content (42). Specifically, the researchers tried to explore tennis-related injuries in terms of the types of injuries, level of expertise, and tournament types throughout the coding process. Although this technique requires subjective assessment, inter-coder reliability aids in the achievement of a systematic examination (43).

### Selection and exclusion criteria for coding and analysis

**Figure 1:**
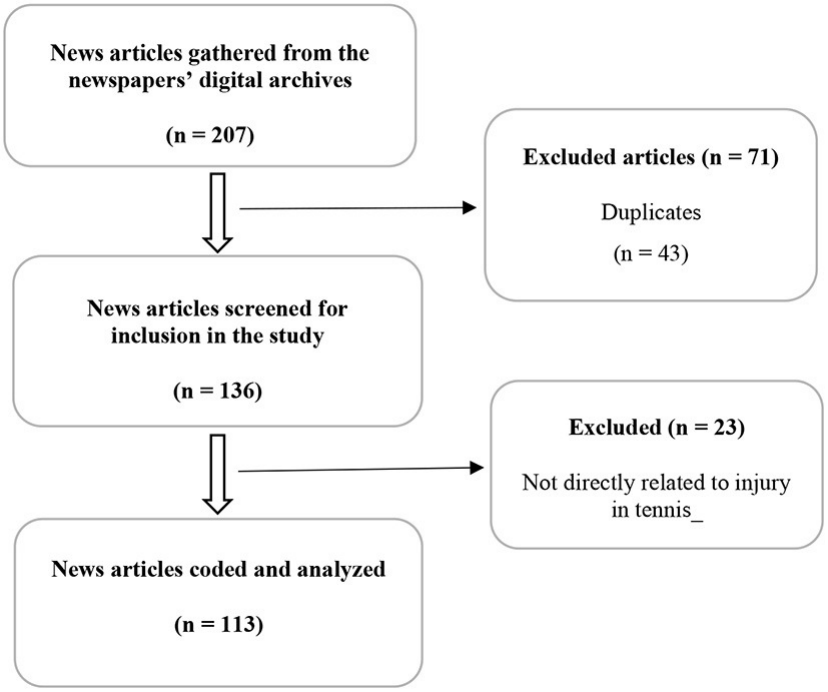
Inclusion and exclusion criteria.

As shown in Figure 1, a total of 207 news reports were generated and screened for inclusion or exclusion from the study. Subsequently, 71 articles were excluded from the study because they are either duplicates or summary updates of existing news articles. In the second step of screening, 23 news articles were also excluded from the study due to inappropriateness. To be included in the sample, each article had to meet six a priori criteria: (1) the article must focus on tennis injury reports; (2) must be straight news or feature story; (3) must be published between January 2015 and September 2020; (4) must be published in the English language; (5) must be an online news article; (6) must be located in the respective digital archives of the selected newspapers. Finally, 113 news reports directly related to injury in tennis were coded and analyzed.

The articles were coded by two PhD candidates who were anonymous to each other. Since two coders were involved in the coding process, Cohen's kappa test was used to assess the inter-coder agreement as proposed in prior research (44). The Cohen's kappa analysis showed an inter-coder agreement of 1.000 concerning players' levels of expertise and types of tournaments as well as 0.961 for injury types and 0.896 for injury status. Cohen's kappa test has been widely accepted as an effective means of ascertaining inter-coder reliability for content analysis (45–47).

### Study variables and their operational definition

Levels of participation or otherwise categorization of players' expertise in tennis are determined by age (48). The Association of Tennis Professionals (ATP) and the Women Tennis Association (WTA) introduced the age eligibility rule to set a limit of age for players' participation in tournaments (wtatennis.com). The rationale for this age-based effect rather than performance could be attributed to the nature of the tennis game as a physically demanding sport. Although young players may have excellent skills and techniques to compete at the highest level, the maximum speed and strength are not fully developed at the teenage age. For instance, players are not permitted to play a full schedule under the WTA until they attend the age of 18 years, while in ATP, the players must be at least 16 years old (49). Therefore, the importance of applying this age restriction may be to protect young players from the risk of burnout which often compels young athletes to early retirement.

In line with the above background, we categorized the players' expertise level based on the players' age, where players aged 8–17 years were categorized as amateurs, whilst those aged 18 years and above were considered as elites, following the recommendation of the previous investigators (40, 41). Hence, we provide the operational definitions of the variables used in the current investigation as follows.

Injury: A physical damage to the body tissues from a tennis match or training resulting in withdrawal or abstinence from the match or training for a day or more.

Fresh injury: A newly sustained physical harm to the body tissues resulting from a tennis match or training.

Recurrent injury: A repetitive injury that occurred at specific locations.

Recovery: Returning to full training or competition.

Elite players: Players above the age of 18 years.

Amateur players: Players below the age of 18 years.

International tournaments: Tournaments organized internationally by the International Tennis Federation.

National/Regional tournaments. Tournaments organized regionally or internally by the tennis regional or country governing body.

### Data analysis

A Chi-square test is primarily designed to aid in the study of categorical data. On the other hand, the Chi-square test takes into account the counted and classified dataset. As a result, the test will not operate with numeric data that are parametric or continuous. It is worth noting that the data set necessary to conduct the Chi-square test should be in the form of frequency, i.e., count datasets, rather than percentages, percentiles, or relative frequency (50). The Chi-square analysis is used in the current study to examine the incidence, exposure, and status of tennis-related injuries as reported by the selected online newspapers. Fundamentally, the test is applied to examine the tennis-related injury occurrences based on expertise levels (elite vs. amateur), tournament types (international vs. national/regional), and injury status (recurrent, recovery, fresh).

The data analysis is carried out sequentially according to the said objectives. To achieve the first objective of the study, i.e., examining the injury occurrences with respect to expertise levels of the players, elite vs. amateur were used as the dependent variables while the types of injuries reported were treated as the independent variables. For the second objective of the study, injury exposure based on tournaments is investigated. The types of tournaments, i.e., international vs. national/regional, were used as dependent variables whilst the various types of injuries were considered as independent variables. For the third objective, we investigated the status of injuries based on tournament types. Here, we used the types of tournaments as dependent variables, whereas the injury status (fresh, recovery or recurrent) was treated as an independent variable. The SPSS statistical software package was utilized in this study, and all the conclusions are made at an alpha (α) level of ≤0.05 using the SPSS Inc., Chicago, IL, USA, 25.0.

## Results

Table 1 projects the distribution of injury exposure with regards to the players' expertise levels. The first column shows the various types of injuries. The second column represents the count and corresponding percentage of injuries sustained by the elite players. Also, the third column depicts the count and percentage of injuries suffered by the amateur players. Whereas, the last column indicates the total counts of specific injuries that affected both the elite and amateur players. It could be observed that the injury occurrences are not limited to only elite or amateur players as demonstrated by the *p*-value of the Chi-square test (*p* > 0.05). As such, both elite and amateur players could be affected by varying injuries. However, it could be seen from the table that elite players are at a higher risk of sustaining injuries (72.26%) as compared with amateur players (27.40%).

**Table 1:** Injury exposure and level of expertise.

**Injury types**	**Expertise level**	
	**Elite F (%)**	**Amateur F (%)**	**Total**
Achilles	3 (60.00)	2 (40.00)	5
Abdominal	1 (100.00)	0 (0.00)	1
Ankle	6 (75.00)	2 (25.00)	8
Arm	3 (100.00)	0 (0.00)	3
Back	7 (77.80)	2 (22.20)	8
Elbow	7 (58.30)	5 (41.70)	12
Foot	1 (50.00)	1 (50.00)	2
Glute	1 (100.00)	0 (0.00)	1
Groin	3 (100.00)	0 (0.00)	3
Hamstring	2 (50.00)	2 (50.00)	4
Hand	1 (100.00)	0 (0.00)	1
Hip	12 (85.70)	2 (14.30)	14
Knee	10 (66.70)	5 (33.30)	15
Leg	1 (100.00)	0 (0.00)	1
Neck	1 (100.00)	0 (0.00)	1
Shoulder	9 (90.00)	1 (10.00)	10
Thigh	4 (66.70)	2 (33.30)	6
Wrist	4 (80.00)	1 (20.00)	5
Unidentified	6 (50.00)	6 (50.00)	12
Overall total (%)	72.60%	27.40%	112

[$\chi_{\left(18\right)}^{2}$ = 16.542; p = 0.555]. F, frequency; (%), percent.

**Figure 2:**
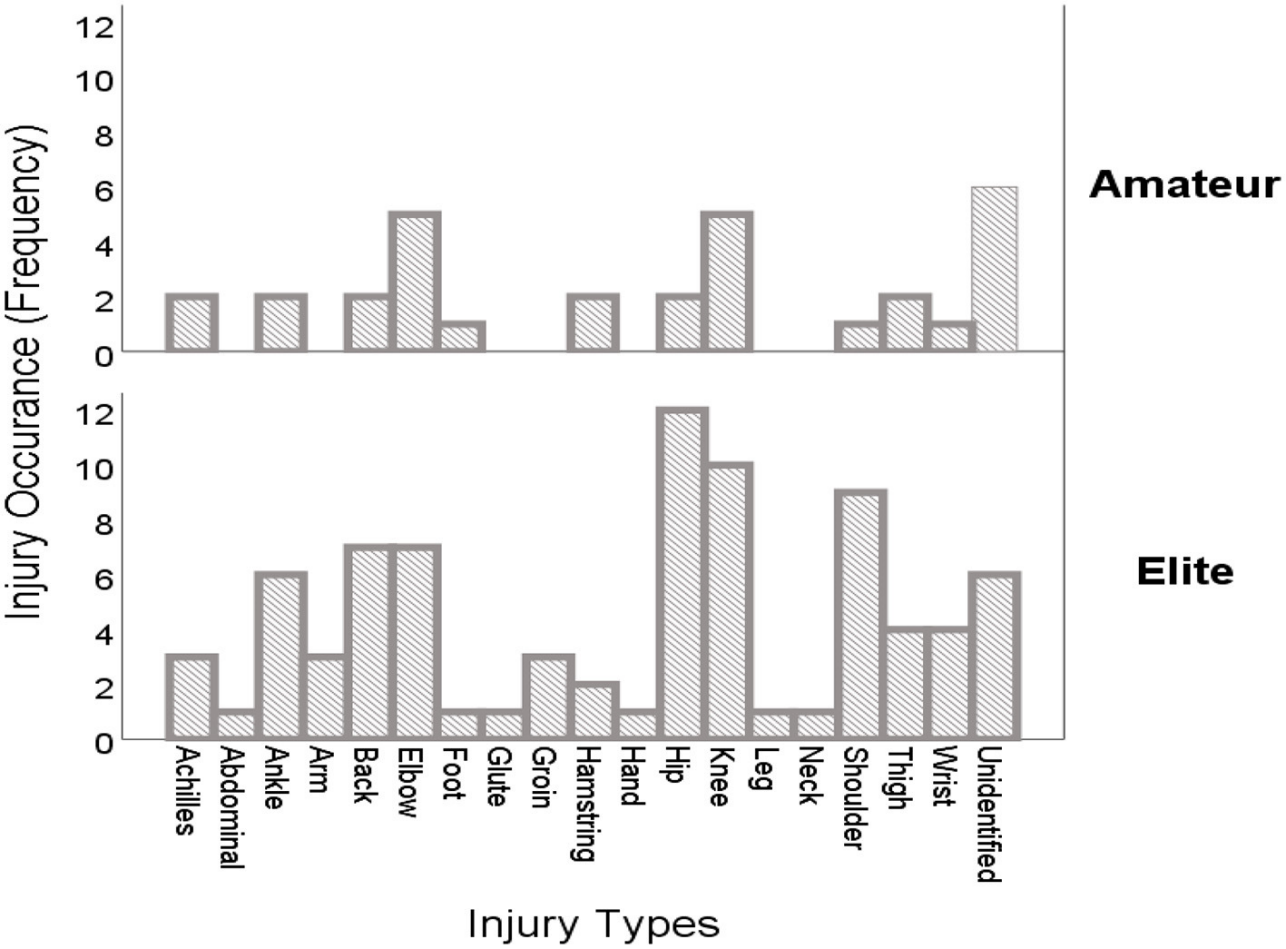
Injury occurrences among elite and amateur players.

Figure 2 displays the graphical illustration of injury occurrences between the elite and amateur players. The y-axis represents injury occurrences (counts) with regards to both amateur and elite players, whilst the x-axis shows the varying types of injuries reported. It can be detected from the figure that specific types of injuries such as abdominal, arm, glute, groin, hand, leg, and neck injuries are especially peculiar to elite players as they were not observed or reported in amateur players. However, some specific injury types such as achille, ankle, back, elbow, foot, hamstring, hip, knee, shoulder, thigh, and wrist injuries are common to both amateur and elite players.

Table 2 presents the incidence of injury status based on the types of tournaments the players engage in. The first column shows the injury status (fresh, recovery, and recurrent). The second column represents the count and corresponding percentage of injuries sustained at international tournaments. Whereas, the third column depicts the count and percentage of injuries suffered by players at national/regional tournaments. Additionally, the fourth column demonstrates the number of unidentified injuries, while the last column indicates the total count of injuries sustained by players with respect to their status. It could be noticed that the injury status is disseminated based on tournament types (*p* > 0.05). Nonetheless, larger amounts of injury incidence within the tournaments are freshly sustained (63), with a relatively high rate of recurrent injury (41). Unfortunately, only a few recoveries were observed (9) with regards9 to the tournaments.

**Table 2:** The prevalence of injury status across tournaments.

**Injury status**	**Types of tournaments**			
	**International F (%)**	**National/regional (%)**	**Unidentified (%)**	**Total**
Fresh	56 (88.90)	3 (4.80)	4 (6.30)	63
Recovery	8 (88.90)	0 (0.00)	1 (11.10)	9
Recurrent	32 (78.00)	3 (7.30)	6 (14.60)	41
Overall total (%)	85.00	5.30	9.70	113

[$\chi_{\left(4\right)}^{2}$ = 3.374; p = 0.497].

**Figure 3:**
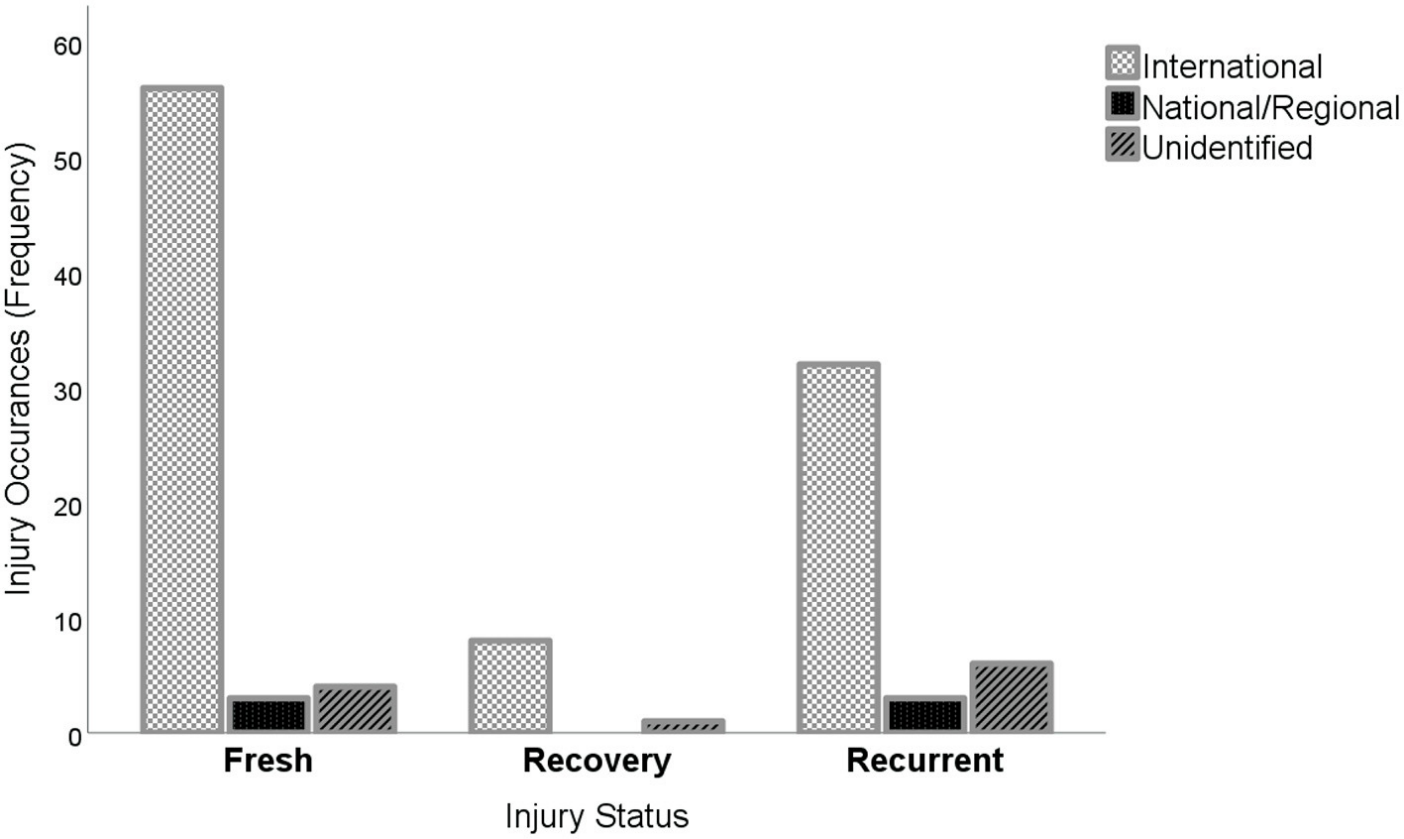
Types of tournaments and injury status.

Figure 3 illustrates the graphical representation of injury status and types of tournaments. It could be observed that a high rate of fresh injury occurrences was reported at international tournaments. However, no recovery rate was observed within national/regional tournaments, whilst a few injuries were unidentified by the media reports.

Table 3 presents the distribution of injury types based on the tournaments organized. The table further shows that the several injuries reported could occur irrespective of the tournament types (*p* > 0.05). Even so, most of the injuries are sustained at international tournaments (85%). However, only 5.30% occurred at national/district tournaments, while 9.70% were unidentified injuries.

**Table 3:** Types of injury exposure at international and national tournaments.

**Injury types**	**Types of tournaments**			
	**International F (%)**	**National/regional F (%)**	**Unidentified**	**Total**
Achilles	5 (100.00)	0 (0.00)	0 (0.00)	5
Abdominal	1 (100.00)	0 (0.00)	0 (0.00)	1
Ankle	6 (75.00)	1 (12.50)	1 (12.50)	8
Arm	2 (66.70)	0 (0.00)	1 (33.30)	3
Back	8 (88.90)	0 (0.00)	1 (11.10)	9
Elbow	11 (91.70)	0 (0.00)	1 (8.30)	12
Foot	2 (100.00)	0 (0.00)	0 (0.00)	2
Glute	1 (100.00)	0 (0.00)	0 (0.00)	1
Groin	2 (66.70)	0 (0.00)	1 (33.30)	3
Hamstr9ing	4 (100.00)	0 (0.00)	0 (0.00)	4
Hand	1 (100.00)	0 (0.00)	0 (0.00)	1
Hip	11 (78.60)	0 (0.00)	3 (21.40)	14
Knee	15 (100.00)	0 (0.00)	0 (0.00)	15
Leg	1 (100.00)	0 (0.00)	0 (0.00)	1
Neck	1 (100.00)	0 (0.00)	0 (0.00)	1
Shoulder	10 (100.00)	0 (0.00)	0 (0.00)	10
Thigh	5 (83.30)	1 (16.70)	0 (0.00)	6
Wrist	5 (100.00)	0 (0.00)	0 (0.00)	5
Unidentified	5 (14.70)	4 (33.30)	3 (25.00)	12
Overall total	85.00%	5.30%	9.70%	113

[$\chi_{\left(36\right)}^{2}$ = 39.393; p = 0.321].

**Figure 4:**
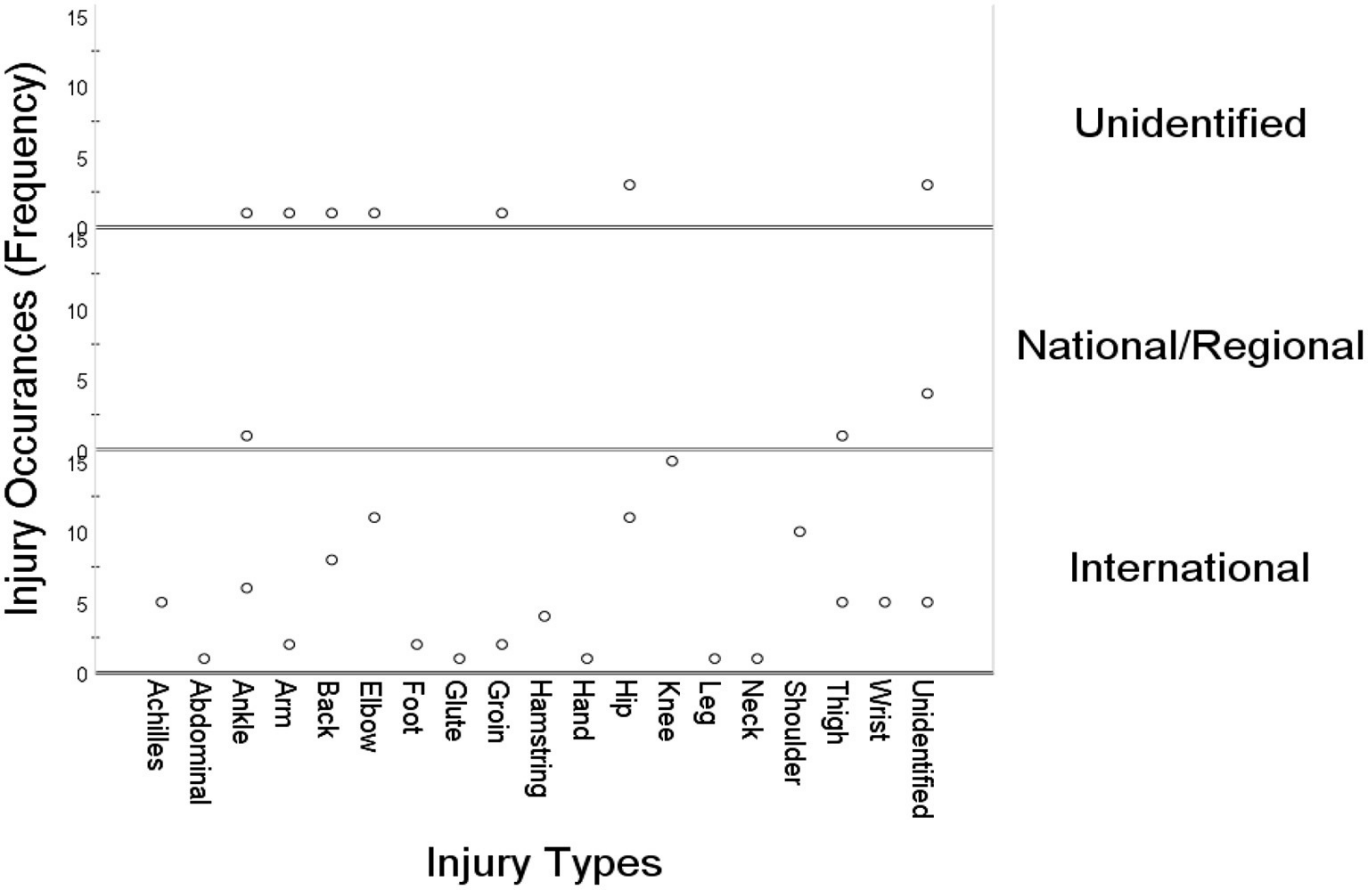
Types of injury occurrences at different tournaments.

Figure 4 highlights the scatter plots of injury distributions based on the tournament types. The y-axis represents the injury occurrences (frequency) with regards to the types of tournaments. The x-axis shows the varying types of injuries reported within a specific tournament. It can be visualized from the figure that different types of injuries are reported with respect to the tournaments. However, most of such injuries are somewhat dominant within international tournaments. The most commonly reported injuries at international tournaments are knee, hip, elbow, and shoulder injuries. Whereas, a few cases of ankle and thigh injuries are observed at national/regional tournaments, others were unidentified.

## Discussion

The purpose of the current investigation was to examine the occurrences and exposure of tennis-associated injuries based on expertise levels and tournament types. A media-based content analysis was employed in the current investigation due to the ability of online-based media content in providing timely and readily available injury-related news to quantify and characterize various injuries to mitigate the problem of data paucity. It is demonstrated from the findings of this study that injury occurrences are distributed across expertise levels (Table 1). However, elite players are at a higher risk of sustaining injuries as opposed to amateur players (Figure 2). Moreover, the prevalence of injury status is distributed based on tournament types (Table 2). Nonetheless, a high rate of fresh injury incidences was reported at international tournaments, whilst no recovery rate was observed within national/regional tournaments, with a few unidentified injuries reported by the media (Figure 3). The findings further demonstrated that several injuries occur irrespective of the tournament types (Table 3). Whereas, most of such injuries, i.e., knee, hip, elbow, and shoulder injuries, are somewhat dominant within international tournaments, a few cases of ankle and thigh injuries were observed at national/regional tournaments with few others unidentified (Figure 4).

The findings of the current investigation revealed that elite players are at a higher risk of sustaining injuries (72.26%) as compared to amateur players (27.40%). This result does not come as a surprise because elite tennis players are occupied with competitions at various tournaments throughout the year, with a tremendous increase in the playing hours. According to the survey conducted in previous research, elite junior players recorded a high incidence of injury over the year, with 41% of the players reporting at least one injury in the preceding year, while 38% were reported to have sustained a second injury within the year (2). Similarly, the authors further reported an association between injury occurrences and the age of tennis players, with older players dominating the chart. Moreover, it has been reported that tennis elite players are attributed to a high chance of sustaining injuries as a result of a congested competition calendar, coupled with high training loads as well as the stress induced by the desire to attain a high-performance level through obtaining a competitive edge (51). Moreover, our study digs deeper into understanding the types of injuries that are most common in elite players. As projected in Figure 3, abdominal, arm, glute, groin, hand, leg, and neck injuries are found to be associated with elite players as opposed to amateur players. This result suggests that injury occurrences in elite players could be multi-faceted, which can affect both muscles and tendon structure. Hence, the nature of injuries may be influenced by several conditions, including the exposure status, i.e., during competition or training as well as the injury mechanisms (recurrent, fresh, or slow-onset) (28).

It was observed from the findings of our study that larger proportions of injury incidence across the tournaments are freshly sustained (63), with relatively high recurrent injury rates (41). Unfortunately, only a few recovery cases were observed (9) with regards to the types of tournaments. This outcome is unanimous with the findings of previous research which revealed that the escalation of injuries over consecutive days translated to a significant decline in performance. Thus, the study postulated that the accumulation of successive matches within a short period, coupled with the lack of adequate recovery time, could escalate the incidence of injury (52). Moreover, other researchers reported that the incessant and rapid rise in training loads might be attributed to a larger incidence in the individualized occurrences of recurrent injury in tennis (53). It could then be inferred that the few proportions of recoveries reported may be related to the tight schedule of tournaments in conformity with a relatively short time of rest between games.

It is evident from the results that the most commonly reported injuries at international tournaments are knee, hip, elbow, and shoulder injuries. It is, therefore, established that the most common tennis-related injuries occur in the lower, trunk, and upper regions, respectively. Tennis is a sport that may be played on a variety of surfaces, including clay, grass, and hard courts. The characteristics of the playing surface may expose players to various forms of injuries. For example, it has been noticed that the ball travels quicker on hard courts, and therefore its speed might cause an additional force to be applied to the upper body (3). Although there is insufficient evidence linking types of injuries and injury incidence to the playing surface, muscle is sensitive to surface stiffness. Therefore, regular play on a variety of surfaces might be associated with lower extremity injury (54, 55). Moreover, it was documented that the prevalence of knee injury in tennis is significantly high as the location is exposed to recurring excessive load (56). Tennis, as opposed to other sports such as soccer, is characterized by sprinting and gliding, where loading primarily affects the lower extremities (57). In addition, tennis does not have a certain fixed duration and hence matches often last numerous hours with many shorts and bursts of energy (2, 3). This unique nature of tennis fosters the occurrence of injuries in the lower, trunk, and upper body regions that are regarded to be greater than in other non-contact sports (58).

Finally, it is essential to highlight that the selected online newspapers in the present study reported more global news on tennis injuries than regional, local, or national news. These findings demonstrate the perceived importance accorded to tennis-related injuries in the selected newspapers. This is because, based on the assumption of the saliency-based technique, the extent of news coverage is considered crucial in defining the level of prominence accorded to the subject being reported (59, 60). Tennis-related injuries in Nigeria garner a lot of media attention because of this notion. However, for information about tennis-related injuries, the selected newspapers rely on news agencies such as Agence France-Presse (AFP), News Agency of Nigeria (NAN), and Reuters, as well as other traditional media such as the British Broadcasting Corporation (BBC) and Cable News Network (CNN).

## Conclusion

The present study examined the incidence and exposure of tennis-associated injuries based on players' levels of expertise and types of tournaments. A media-based content analysis was employed to achieve the purpose of this study due to the ability of the online-based media content in providing timely and readily available injury-related news for the quantification and characterization of various injuries to mitigate the problem of data scarcity. It is shown in the present investigation that the occurrences of tennis-associated injuries are disseminated across expertise levels, with knee, hip, elbow, and shoulder injuries being highly prevalent in both elite and amateur players. However, it is observed that elite players recorded a staggering percentage of injury-related problems as compared with amateur players. Conversely, the injury status is distributed amongst tournament types, with fresh injuries dominating, followed by recurrent and recovery injuries. The findings further demonstrated that the injury types are sustained regardless of tournament types. Nonetheless, most of the injuries are sustained at international tournaments. Also, it could be deduced from the findings of this investigation that elite players are more likely to sustain injuries compared with amateur players. Also, the highly frequent tennis-associated injuries happen in the lower, trunk, and upper parts of the body, respectively. A larger proportion of the reported tennis injuries are fresh and recurrent with a few recoveries. The international tennis tournaments are highly attributed to injury occurrences as opposed to national/regional tournaments. The application of the media-based content analysis technique is non-trivial in projecting injury-related problems that could be used to facilitate the development of an injury index peculiar to the tennis sport for prompt intervention.

## Recommendations

The most frequently reported injuries amongst the players at international as well as national/regional tournaments are knee, hip, elbow, shoulder, ankle, and thigh injuries. To mitigate the occurrences of injury in these regions, specific injury prevention exercises that consist of vastus medialis oblique muscles exercises, band-related exercise, dynamic and regulated lounges coupled with one-legged squats could assist in strengthening the knee. The clamshells and reversed clamshells, hip abduction, and adduction, plyometric lateral stepovers, and elastic tubing kicks could aid in preventing hip injuries. Exercises for the elbow could aim toward increasing the strength and wrist and forearm musculature. Hence, flexor-extensor wrist curls, forearm pronation, and supination as well as the application of a counterbalanced weight or gripping dumbbell at one end in the process of isolated movement of radial and ulnar deviation of the wrist could be used to address the elbow injury. For the shoulder, Jobe rotator cuff exercises, side-lying external rotation, prone extension, prone horizontal abduction, prone external rotation, shoulder external rotation (neutral tubing), shoulder external rotation 90, abduction (tubing) as well as Shoulder 90/90 prone plyometrics exercises could be beneficial. Towel curl, toe pull, standing heel raise, and golf ball roll could help in tackling ankle injuries. Exercises such as lying leg extension, standing hamstring stretch, static quadriceps stretch as well as passive quadriceps stretch could be useful for the thigh-related injuries (42, 61). These sets of exercises could be beneficial to both the athletes and the coaching staff as they may serve as an intervention strategy to prolong and safeguard the players' careers as well as improve their performance.

## Practical implication of the study

Identification of the frequent injury sites in tennis players is a non-trivial task as it could serve as a means of injury prevention strategy through targeted training and exercise by the strength and conditioning experts at the specific location. In the current investigation, we identified the most prevalent injuries affecting both elite and amateur tennis players. We also determined the kinds of tournaments that highly triggered injury occurrences among tennis players. We proposed specific injury prevention exercises/training for the highly prone injury locations affecting the players. The exercises, as well as the specific pieces of training proposed, could guide coaches, strength and conditioning experts, and trainers to restructure and develop a suitable training routine that can address the injury occurrences and reduce the risk of injury in players during practice and competitions at various tournaments.

## Limitation of the study

The current study is subject to certain limitations. First, only Nigerian electronic newspapers were considered in this study. Second, the data were gathered from selected newspaper outlets, and the analysis was based on a relatively small number of reports, representing larger tennis-related injury occurrences. Third, although the study relied on newspapers as they are proven to be verified sources of injury data, the investigation was based on a content analysis of news reports which could not consider other factors, such as direct responses from the athletes or the respective medical teams. Moreover, the investigation relied on media content published in English *per se*, which inevitably excludes tennis injury reports produced in other languages. Finally, it is important to ascertain the efficacy of specific training routines for injury prevention in this sport. Nonetheless, this is beyond the scope of the current study. Hence, it is proposed that future research could consider the aforementioned variables for the analysis of injury in tennis sport.

## Data availability statement

The raw data supporting the conclusions of this article will be made available by the authors, without undue reservation.

## Author contributions

RM and IH: conceptualization and formal analysis. RM, IH, MA, and ML: methodology and data curation. AA and IH: software and visualization. NA and AA: validation. MA and ML: investigation. AA, IH, and NA: resources. AA, IH, and RM: writing—original draft preparation and project administration. NA, MA, ML, and RM: writing—review and editing. NA, MA, and ML: supervision. All authors have read and agreed to the published version of the manuscript. All authors contributed to the article and approved the submitted version.

## Conflict of interest

The authors declare that the research was conducted in the absence of any commercial or financial relationships that could be construed as a potential conflict of interest.

## Publisher's note

All claims expressed in this article are solely those of the authors and do not necessarily represent those of their affiliated organizations, or those of the publisher, the editors and the reviewers. Any product that may be evaluated in this article, or claim that may be made by its manufacturer, is not guaranteed or endorsed by the publisher.

## Appendix

**Appendix 1:** Codebook.

**Variable**	**Code1**	**Categories**	**Code2**	**Description**	**Exemplary quotes**
Levels of expertise	LE	Amateur Level of Expertise	ALE	Players aged 8 to 17 years are categorized as amateurs based on the age eligibility rule set by ATP and WTA as well as previous research	The 10-year-old...twisted her ankle while featuring in a tournament in London (ThisDay Newspaper, April 24, 2017)
		Elite Level of Expertise	ELE	Players aged 18 years and above are categorized as elites based on the age eligibility rule set by ATP and WTA as well as previous research.	The 29-year-old is still coming back from a wrist injury suffered in 2017 (Vanguard Newspaper, May 21, 2019)
Types of tournaments	TT	International Tennis Tournament	ITT	Tennis-related injuries reported at a tournament in a global context that might be of interest to a global audience, for example, the Brisbane International Tennis Tournament	Nadal pulled out of the Brisbane International warmup with a thigh strain (Vanguard Newspaper, January 11, 2019)
		Regional Tennis Tournament	NTT	Tennis-related injuries reported at a tournament in a regional context that might not be of interest to other regions, such as the Central Africa Junior Tennis Championships	An injury she sustained at the ITF/CAT West and Central Africa Junior Tennis Championships (AJC) (ThisDay Newspaper, April 24, 2017)
		National Tennis Tournament	RTT	Tennis-related injuries reported at a tournament in a national context that might not be of interest to other nations, for instance, the Nigeria Oil and Gas Industry Games	That led to his retirement on account of injury [at] the 2018 Nigeria Oil and Gas Industry Tournament (ThisDay Newspaper, February 24, 2018)
		Unidentified Tennis Tournament	UTT	Any tennis-related injury report in which the tournament's name is not clearly mentioned in the article	Twelve-time Grand Slam champion Novak Djokovic will miss the rest of the season with an elbow injury (Punch Newspaper, July 26, 2017)
Injury status	IS	Fresh injury	FRI	Any tennis-related injury sustained by an athlete at a particular site that has not been previously injured at a given tournament	Serena Williams withdraws from Italian Open with new injury (Vanguard Newspaper, May 14, 2019)
		Recovery injury	RCI	Any tennis-related injury report involving an account of a previously injured athlete who regained competitive ability to participate in a given tournament	World number one Simona Halep...has recovered and is feeling fit heading into the new season (Punch Newspaper, January 6, 2020)
		Recurrent Injury	RRI	Any previously sustained tennis-related injury that occurred again at the same location after a player's return to full participation	Novak Djokovic has said that a recurring wrist injury has “taken its toll” after a “very busy and active” year (ThisDay Newspaper, August 11, 2016)
Injury types	IT	Achilles Injury	ACI	As mentioned in the articles	Serena Williams has withdrawn from the French Open because of an Achilles injury (ThisDay Newspaper, October 1, 2020)
		Abdominal Injury	ABI	As mentioned in the articles	She [Osaka] suffered an abdominal injury in Stuttgart last month (Vanguard Newspaper, May 21, 2019)
		Ankle injury	AKI	As mentioned in the articles	Andy Murray has...pulled out of the China Open with an ankle complaint (Punch Newspaper, September 30, 2018)
		Arm Injury	AMI	As mentioned in the articles	Citing left arm soreness, Maria Sharapova has withdrawn from the Bank of the West Classic in Stanford (The Nation Newspaper, August 2, 2017)
		Back Injury	BCI	As mentioned in the articles	Serena Williams' first U.S. Open tune-up ended in dramatic fashion as the tearful American was forced to retire with a back injury on Sunday (The Nation Newspaper, August 12, 2019)
		Elbow Injury	ELI	As mentioned in the articles	Novak Djokovic will miss the rest of the season with an elbow injury (Punch Newspaper, July 26, 2017)
		Foot Injury	FTI	As mentioned in the articles	A right foot stress fracture halted that momentum, forcing her to withdraw from the US Open (ThisDay Newspaper, September 11, 2017)
		Glute Injury	GLI	As mentioned in the articles	Canada's Milos Raonic has pulled out of the US Open with a glute injury (Punch Newspaper, August 26, 2019)
		Groin Injury	GRI	As mentioned in the articles	...because of a groin injury that has left him unable to practice on the court (Vanguard Newspaper, December 11, 2019)
		Hamstring Injury	HMI	As mentioned in the articles	Japan's Naomi Osaka has withdrawn from Saturday's Western and Southern Open final due to a left hamstring injury (Vanguard Newspaper, August 29, 2020)
		Hand Injury	HDI	As mentioned in the articles	Federer...had been suffering since the summer with a hand injury (Punch Newspaper, October 22, 2018)
		Hip Injury	HPI	As mentioned in the articles	Unfortunately, I have withdrawn from Doha and Dubai with a minor hip injury said Barty (Punch Newspaper, February 12, 2019)
		Knee Injury	KNI	As mentioned in the articles	Federer has...missed the majority of the 2016 season with a knee injury (Vanguard Newspaper, June 10, 2020)
		Leg Injury	LGI	As mentioned in the articles	Wozniacki withdraws from Washington Open with a leg injury (Punch Newspaper, July 31, 2018)
		Neck Injury	NKI	As mentioned in the articles	Elina Svitolina, who was hampered by pain in her neck (ThisDay Newspaper, January 24, 2019)
		Shoulder Injury	SDI	As mentioned in the articles	A nagging shoulder injury forced the world number one to retire from his fourth-round match (Vanguard Newspaper, September 1, 2019)
		Thigh Injury	TGI	As mentioned in the articles	Quadri suffered a thigh injury during the Tokyo 2020 Olympic qualifying tournament in Tunisia, which saw him withdraw from his last qualification match (Punch Newspaper, March 5, 2020)
		Wrist Injury	WRI	As mentioned in the articles	Rafa Nadal withdrew from the French Open with a left wrist injury (ThisDay Newspaper, June 4, 2016)
		Unidentified Injury	UFI	Tennis-related injury reports in which specific injury locations have not been clearly stated	Murray retired from the Wimbledon during his quarter-final clash against Tomas Berdych with an injury (ThisDay Newspaper, July 13, 2017)
